# Crystal structure of serotonin

**DOI:** 10.1107/S2056989022002559

**Published:** 2022-03-10

**Authors:** Marilyn Naeem, Andrew R. Chadeayne, James A. Golen, David R. Manke

**Affiliations:** a University of Massachusetts Dartmouth, 285 Old Westport Road, North Dartmouth, MA 02747, USA; b CaaMTech, Inc., 58 East Sunset Way, Suite 209, Issaquah, WA 98027, USA

**Keywords:** crystal structure, serotonin, tryptamine, indole, free base

## Abstract

The title compound, serotonin or 5-hy­droxy­tryptamine (5-HT) [systematic name: 3-(2-amino­eth­yl)-1*H*-indol-5-ol], C_10_H_12_N_2_O, has one mol­ecule in the asymmetric unit. The conformation of the ethyl­amino side chain is *gauche*–*gauche* [C_a_—C_a_—C_m_—C_m_ and C_a_—C_m_—C_m_—N (a = aromatic, m = methyl­ene) torsion angles = −64.2 (3) and −61.9 (2)°, respectively]. In the crystal, the mol­ecules are linked into a three-dimensional network by N—H⋯O and O—H⋯N hydrogen bonds.

## Abstract 2

The crystal structure of the free base of the ubiquitous neurotransmitter serotonin is reported for the first time.

## Chemical context

Serotonin, C_10_H_12_N_2_O, systematic name 3-(2-amino­eth­yl)-1*H*-indol-5-ol, is the primary neurotransmitter in humans, regulating mood, anxiety and happiness (Young & Leyton, 2002 ▸). While it is best known for its role in the central nervous system, serotonin is found throughout the human body and impacts a wide array of bodily functions. Roughly ninety-five percent of the body’s serotonin is actually found in the gastrointestinal tract, where it regulates intestinal movement (Berger *et al.*, 2009 ▸). Serotonin is produced in the human body through biosynthesis from the essential amino acid tryptophan (Fitzpatrick, 1999 ▸), and broken down by mono­amine oxidase to generate 5-hy­droxy­indole­acetic acid. As such, mono­amine oxidase inhibitors and other compounds that increase serotonin concentration have been used to treat depression (Suchting *et al.*, 2021 ▸). 
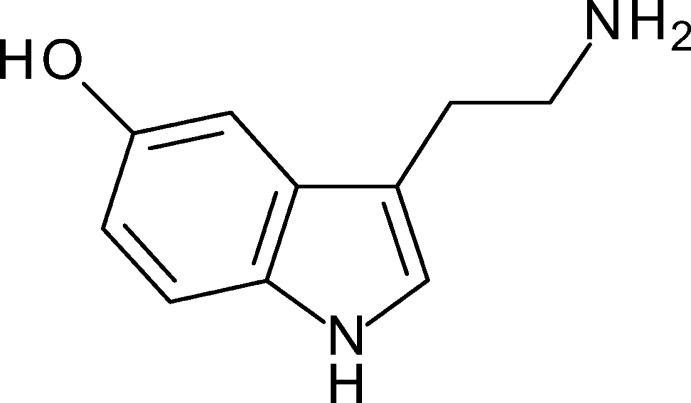


Serotonin is not unique to humans, but is found throughout life on Earth including all bilateral animals, where it also functions as a neurotransmitter (Bacqué-Cazenave *et al.*, 2020 ▸). It is found in plants, notably in seeds, where serotonin stimulates the digestive tract of animals, leading to excretion of the seeds (Akula *et al.*, 2011 ▸). Serotonin and related tryptamines are well known to be present in a number of fungi (Tyler, 1958 ▸; Sherwood *et al.*, 2020 ▸). A variety of related tryptamines found in plants, fungi, and toads, which are active at serotonin receptors, have garnered significant attention as psychedelic drugs to treat mood disorders including anxiety, depression, and addiction (Carhart-Harris & Goodwin, 2017 ▸). Serotonin was discovered by Vittorio Erspaner in 1935, characterized as 5-hy­droxy­tryptamine (5-HT) in 1949 by Rapport, and synthesized by Upjohn pharmaceutical in 1951 (Whitaker-Azmitia, 1999 ▸). Despite the simplicity of its structure and universally recognized biological significance, the single-crystal structure of pure free base serotonin has never been reported. Herein, we report this structure to fill in the gap from the scientific record.

## Structural commentary

**Figure 1:**
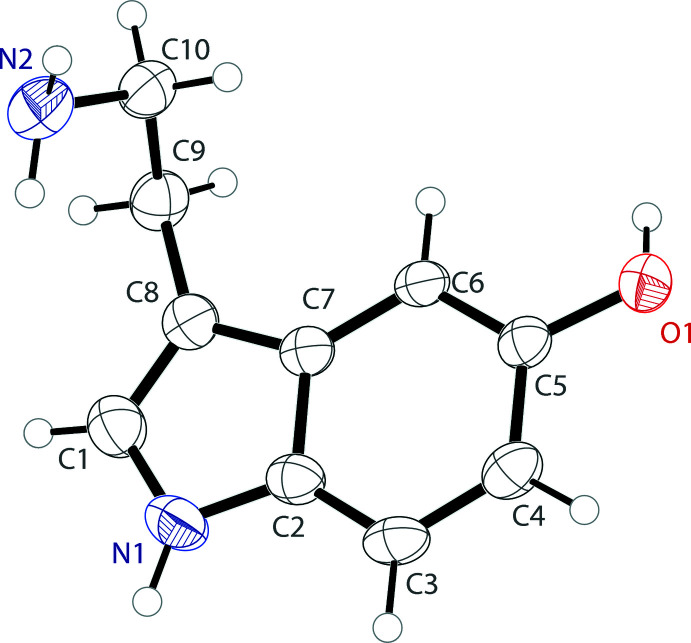
The mol­ecular structure of serotonin free base showing the atomic labeling. Displacement ellipsoids are drawn at the 50% probability level.

Serotonin or 5-hy­droxy­tryptamine (5-HT) is an indolamine with a 5-hy­droxy substitution. In the solid state, serotonin crystallizes with one mol­ecule in the asymmetric unit (Fig. 1 ▸) in the chiral space group *P*2_1_2_1_2_1_. The 5-hy­droxy­indole fused-ring unit is almost planar with the non-hydrogen atoms showing an r.m.s. deviation from planarity of 0.030 Å. The ethyl­amino arm is turned away from the indole ring, with a C7—C8—C9—C10 torsion angle of −64.2 (3)°. The ethyl­amino arm itself turns back toward the indole ring with a C8—C9—C10—N2 torsion angle of −61.9 (2)°.

## Supra­molecular features

**Figure 2:**
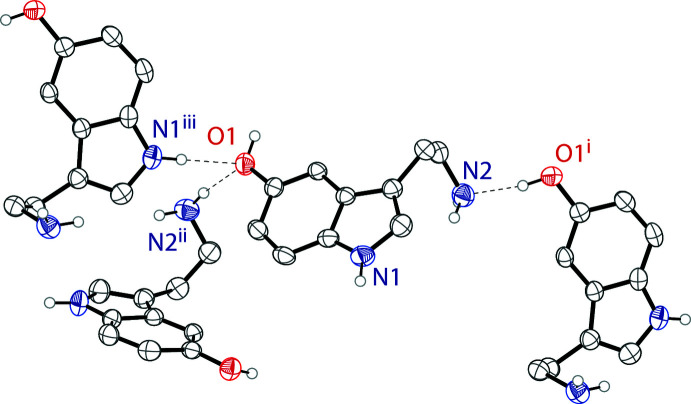
The different hydrogen-bonding inter­actions between the serotonin mol­ecules. Hydrogen atoms not involved in hydrogen bonding are omitted for clarity. Symmetry codes: (i) 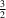 − *x*, 1 − *y*, 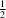 + *z* (ii) 2 − *x*, − 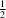 + *y*, 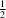 − *z* (iii) 3/2 − x, −y, − 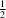 + *z*.

**Figure 3:**
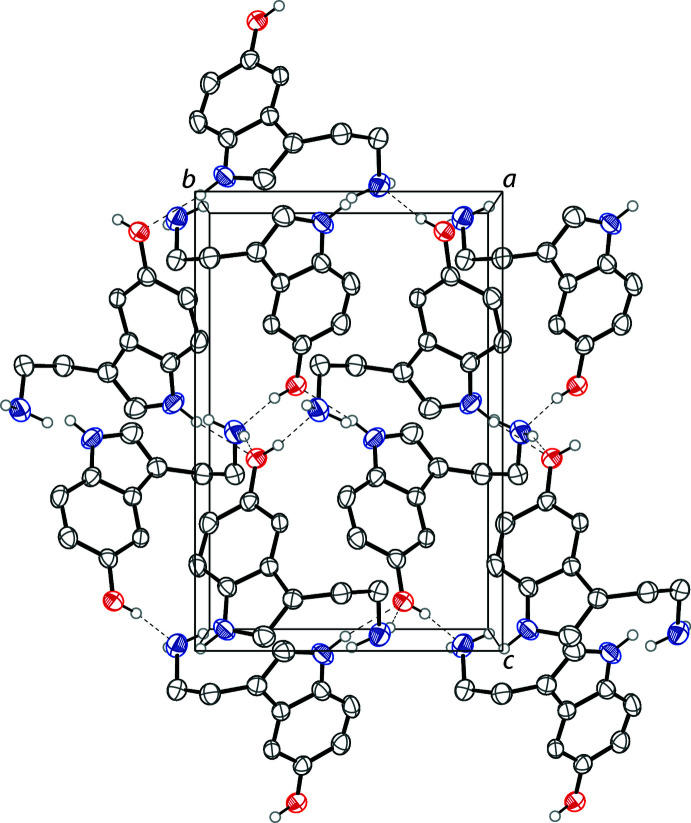
The crystal packing of serotonin free base viewed along the *a*-axis. Hydrogen bonds are shown as dashed lines. Hydrogen atoms not involved in hydrogen bonds are omitted for clarity.

In the crystal, the serotonin mol­ecules are linked by a series of hydrogen bonds that produce a three-dimensional network in the solid state. The hy­droxy groups form hydrogen bonds to the amine N atoms on an adjacent serotonin mol­ecules forming O1—H1⋯N2 hydrogen bonds. The indole N atoms form hydrogen bonds to the hy­droxy groups of adjacent serotonin mol­ecules through N1—H1*A*⋯O1 hydrogen bonds. Half of the amine H atoms link to the hy­droxy groups of nearby mol­ecules through N2—H2*B*⋯O1 hydrogen bonds. There are no observed π–π stacking inter­actions. Fig. 2 ▸ outlines the hydrogen bonds, which are detailed in Table 1 ▸. The crystal packing of serotonin is shown in Fig. 3 ▸.

## Database survey

The previous structural reports of serotonin are all complex mixtures containing serotonin in its C_10_H_13_N_2_O^+^ cationic form. These include the creatine sulfate monohydrate (Karle *et al.*, 1965 ▸: Cambridge Structural Database refcode HTRCRS), the hydrogen oxalate salt (Amit *et al.*, 1978 ▸: SERHOX), the hydro­adipate salt (Rychkov *et al.*, 2013 ▸: VIKWIX), the picrate monohydrate (Thewalt & Bugg, 1972 ▸: SERPIC) and two compounds where it is co-crystallized with 1,3,6,8-tetra­sulfonato­pyrene (Feng *et al.*, 2017 ▸: RAWDIF, RAWDOL). The two most closely reported free-base structures to serotonin are the natural product bufotenine, 5-hy­droxy-*N*,*N*-di­methyl­tryptamine (Falkenberg, 1972 ▸: BUFTEN) and 5-meth­oxy­tryptamine (Quarles *et al.*, 1974 ▸: MXTRYP). 5-Meth­oxy­tryptamine has also been reported as its picrate (Nagata *et al.*, 1995 ▸: ZILMIQ) and chloride (Pham *et al.*, 2021 ▸: CCDC 2106050) salts. The free base reported here shows the ethyl­amino arm turned away from the indole plane. The majority of the cationic tryptamine structures show ethyl­amino arms that are nearly in-plane with the indole ring. Only the picrate salt has a structure similar to that of the title compound, showing an ethyl­amino arm turned similarly away from the indole ring. The torsion angles associated with the ethyl­amino arms of the different structures are summarized in Table 2 ▸.

## Synthesis and crystallization

Single crystals suitable for X-ray diffraction studies were grown from the slow evaporation of a tetra­hydro­furan solution of a commercial sample of serotonin free base (Chem-Impex).

## Refinement

Crystal data, data collection and structure refinement details are summarized in Table 3 ▸. Hydrogen atoms H1, H1*A*, H2*A* and H2*B* were found from a difference-Fourier map and were refined isotropically, using DFIX restraints with an N—H(indole) distance of 0.87 (1) Å, N—H(amine) distances of 0.90 (1) Å, and an O—H distance of 0.86 (1) Å. Isotropic displacement parameters were set to 1.2 *U*_eq_ of the parent nitro­gen atoms and 1.5 *U*_eq_ of the parent oxygen atom. All other hydrogen atoms were placed in calculated positions with C—H = 0.93 Å (*sp* ^2^) or 0.97 Å (*sp* ^3^). Isotropic displacement parameters were set to 1.2 *U*_eq_ of the parent carbon atoms. The absolute structure of the crystal chosen for data collection was indeterminate in the present refinement.

## Supplementary Material

Crystal structure: contains datablock(s) I, global. DOI: 10.1107/S2056989022002559/hb8014sup1.cif

Structure factors: contains datablock(s) I. DOI: 10.1107/S2056989022002559/hb8014Isup2.hkl

Supporting information file. DOI: 10.1107/S2056989022002559/hb8014Isup3.cml

CCDC reference: 2156646

Additional supporting information:  crystallographic information; 3D view; checkCIF report

## Figures and Tables

**Table 1:** Hydrogen-bond geometry (Å, °)

*D*—H⋯*A*	*D*—H	H⋯*A*	*D*⋯*A*	*D*—H⋯*A*
O1—H1⋯N2^i^	0.88 (1)	1.77 (1)	2.636 (2)	170 (3)
N1—H1*A*⋯O1^ii^	0.88 (1)	2.10 (1)	2.967 (2)	169 (2)
N2—H2*B*⋯O1^iii^	0.91 (1)	2.19 (1)	3.092 (3)	168 (2)

Symmetry codes: (i) 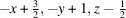 ; (ii) 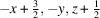 ; (iii) 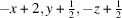 .

**Table 2:** Torsion angles of the ethyl­amino arms of different serotonin structures (our atom-numbering scheme)

	Space group	C7—C8—C9—C10	C8—C9—C10—N2	Reference
5-HT free base	*P*2_1_2_1_2_1_	−64.2 (3)	−61.9 (2)	This work
HTRCRS	*C*2/*c*	166.7	172.6	Karle *et al.* (1965 ▸)
SERHOX	*P*2_1_/*n*	171.7	179.7	Amit *et al.* (1978 ▸)
SERPIC	*P*2_1_/*c*	67.5	66.6	Thewalt & Bugg (1972 ▸)
VIKWIX	*P* 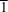	178.7	177.2	Rychkov *et al.* (2013 ▸)
RAWDIF	*Pca*2_1_	177.8	177.6	Feng *et al.* (2017 ▸)
RAWDO*L* ^*a*^	*Cc*	178.7	175.1	Feng *et al.* (2017 ▸)
RAWDO*L* ^*b*^	*Cc*	102	43	Feng *et al.* (2017 ▸)

Notes: (*a*, *b*) RAWDOL contains two mol­ecules in the asymmetric unit. The *b* mol­ecule is probably disordered and the geometrical data are less certain.

**Table 3:** Experimental details

Crystal data	
Chemical formula	C_10_H_12_N_2_O
*M*_r_	176.22
Crystal system, space group	Orthorhombic, *P*2_1_2_1_2_1_
Temperature (K)	297
*a*, *b*, *c* (Å)	8.2248 (6), 8.7542 (6), 13.0712 (10)
*V* (Å^3^)	941.15 (12)
*Z*	4
Radiation type	Mo *K*α
μ (mm^−1^)	0.08
Crystal size (mm)	0.18 × 0.10 × 0.02

Data collection	
Diffractometer	Bruker D8 Venture CMOS
Absorption correction	Multi-scan (*SADABS*; Bruker, 2018 ▸)
*T*_min_, *T*_max_	0.711, 0.745
No. of measured, independent and observed [*I* > 2σ(*I*)] reflections	25138, 1783, 1590
*R*_int_	0.052
(sin θ/λ)_max_ (Å^−1^)	0.610

Refinement	
*R*[*F* ^2^ > 2σ(*F* ^2^)], *wR*(*F* ^2^), *S*	0.030, 0.073, 1.05
No. of reflections	1783
No. of parameters	134
No. of restraints	4
H-atom treatment	H atoms treated by a mixture of independent and constrained refinement
Δρ_max_, Δρ_min_ (e Å^−3^)	0.13, −0.13
Absolute structure	Flack *x* determined using 609 quotients [(*I* ^+^)-(*I* ^-^)]/[(*I* ^+^)+(*I* ^-^)] (Parsons *et al.*, 2013 ▸)
Absolute structure parameter	−1.2 (6)

Computer programs: *APEX3* (Bruker, 2018 ▸), *SAINT* (Bruker, 2018 ▸), *SHELXT2014* (Sheldrick, 2015*a* ▸), *SHELXL2018* (Sheldrick, 2015*b* ▸), *OLEX2* (Dolomanov *et al.*, 2009 ▸), *publCIF* (Westrip, 2010 ▸).

## supplementary crystallographic information

### Crystal data

**Table:**

C_10_H_12_N_2_O	*D*_x_ = 1.244 Mg m^−^^3^
*M**_r_* = 176.22	Mo *K*α radiation, λ = 0.71073 Å
Orthorhombic, *P*2_1_2_1_2_1_	Cell parameters from 6263 reflections
*a* = 8.2248 (6) Å	θ = 3.1–25.4°
*b* = 8.7542 (6) Å	µ = 0.08 mm^−^^1^
*c* = 13.0712 (10) Å	*T* = 297 K
*V* = 941.15 (12) Å^3^	Block, colourless
*Z* = 4	0.18 × 0.10 × 0.02 mm
*F*(000) = 376	

### Data collection

**Table:**

Bruker D8 Venture CMOS diffractometer	1590 reflections with *I* > 2σ(*I*)
φ and ω scans	*R*_int_ = 0.052
Absorption correction: multi-scan (SADABS; Bruker, 2018)	θ_max_ = 25.7°, θ_min_ = 2.8°
*T*_min_ = 0.711, *T*_max_ = 0.745	*h* = −10→10
25138 measured reflections	*k* = −10→10
1783 independent reflections	*l* = −15→15

### Refinement

**Table:**

Refinement on *F*^2^	Hydrogen site location: mixed
Least-squares matrix: full	H atoms treated by a mixture of independent and constrained refinement
*R*[*F*^2^ > 2σ(*F*^2^)] = 0.030	*w* = 1/[σ^2^(*F*_o_^2^) + (0.0372*P*)^2^ + 0.1115*P*] where *P* = (*F*_o_^2^ + 2*F*_c_^2^)/3
*wR*(*F*^2^) = 0.073	(Δ/σ)_max_ < 0.001
*S* = 1.05	Δρ_max_ = 0.13 e Å^−^^3^
1783 reflections	Δρ_min_ = −0.13 e Å^−^^3^
134 parameters	Absolute structure: Flack *x* determined using 609 quotients [(*I*^+^)-(*I*^-^)]/[(*I*^+^)+(*I*^-^)] (Parsons *et al.*, 2013)
4 restraints	Absolute structure parameter: −1.2 (6)

### Special details

**Table:**

Geometry. All esds (except the esd in the dihedral angle between two l.s. planes) are estimated using the full covariance matrix. The cell esds are taken into account individually in the estimation of esds in distances, angles and torsion angles; correlations between esds in cell parameters are only used when they are defined by crystal symmetry. An approximate (isotropic) treatment of cell esds is used for estimating esds involving l.s. planes.

### Fractional atomic coordinates and isotropic or equivalent isotropic displacement parameters (Å^2^)

**Table:**

	*x*	*y*	*z*	*U*_iso_*/*U*_eq_	
O1	0.91964 (18)	0.18734 (16)	0.08462 (10)	0.0398 (4)	
H1	0.868 (3)	0.263 (2)	0.054 (2)	0.078 (9)*	
N1	0.6390 (2)	0.0903 (2)	0.45983 (14)	0.0445 (5)	
H1A	0.633 (3)	0.012 (2)	0.5016 (15)	0.051 (7)*	
N2	0.7279 (2)	0.6020 (2)	0.47447 (15)	0.0458 (5)	
H2A	0.725 (3)	0.5092 (18)	0.5030 (18)	0.050 (7)*	
H2B	0.8312 (19)	0.638 (3)	0.465 (2)	0.072 (9)*	
C1	0.5533 (3)	0.2238 (2)	0.47087 (16)	0.0433 (5)	
H1B	0.487070	0.246998	0.526412	0.052*	
C2	0.7237 (2)	0.0960 (2)	0.36910 (15)	0.0343 (4)	
C3	0.8303 (2)	−0.0077 (2)	0.32446 (16)	0.0388 (5)	
H3	0.856712	−0.098830	0.357097	0.047*	
C4	0.8956 (2)	0.0283 (2)	0.23057 (16)	0.0375 (5)	
H4	0.967797	−0.039127	0.199600	0.045*	
C5	0.8552 (2)	0.1654 (2)	0.18068 (14)	0.0316 (4)	
C6	0.7543 (2)	0.27075 (19)	0.22632 (14)	0.0304 (4)	
H6	0.731045	0.362796	0.193965	0.036*	
C7	0.6874 (2)	0.2373 (2)	0.32214 (14)	0.0300 (4)	
C8	0.5785 (2)	0.3173 (2)	0.38953 (14)	0.0349 (4)	
C9	0.5130 (2)	0.4756 (2)	0.37352 (17)	0.0391 (5)	
H9A	0.434413	0.497950	0.426806	0.047*	
H9B	0.456668	0.479381	0.308368	0.047*	
C10	0.6446 (3)	0.5971 (2)	0.37477 (16)	0.0412 (5)	
H10A	0.723268	0.575700	0.321372	0.049*	
H10B	0.596386	0.696063	0.360712	0.049*	

### Atomic displacement parameters (Å^2^)

**Table:**

	*U*^11^	*U*^22^	*U*^33^	*U*^12^	*U*^13^	*U*^23^
O1	0.0473 (8)	0.0370 (8)	0.0351 (7)	0.0049 (7)	0.0058 (6)	−0.0021 (6)
N1	0.0536 (11)	0.0411 (10)	0.0390 (10)	0.0009 (9)	0.0040 (9)	0.0125 (8)
N2	0.0465 (11)	0.0406 (10)	0.0502 (12)	0.0012 (9)	−0.0064 (9)	−0.0055 (9)
C1	0.0441 (11)	0.0456 (13)	0.0402 (11)	0.0009 (10)	0.0064 (9)	0.0025 (9)
C2	0.0378 (10)	0.0309 (9)	0.0342 (10)	−0.0019 (8)	−0.0068 (9)	0.0044 (8)
C3	0.0434 (11)	0.0268 (9)	0.0461 (11)	0.0031 (8)	−0.0094 (10)	0.0058 (8)
C4	0.0366 (10)	0.0325 (10)	0.0435 (11)	0.0061 (8)	−0.0055 (9)	−0.0036 (9)
C5	0.0322 (9)	0.0299 (9)	0.0328 (9)	−0.0033 (7)	−0.0037 (8)	−0.0034 (8)
C6	0.0360 (9)	0.0230 (8)	0.0323 (9)	−0.0004 (8)	−0.0052 (8)	0.0010 (7)
C7	0.0304 (9)	0.0282 (9)	0.0315 (9)	−0.0012 (7)	−0.0060 (7)	0.0001 (7)
C8	0.0348 (9)	0.0347 (10)	0.0353 (10)	0.0004 (8)	−0.0024 (9)	−0.0004 (8)
C9	0.0377 (10)	0.0390 (11)	0.0406 (11)	0.0078 (9)	−0.0006 (9)	−0.0009 (9)
C10	0.0493 (12)	0.0340 (10)	0.0404 (11)	0.0045 (9)	−0.0001 (10)	−0.0030 (9)

### Geometric parameters (Å, º)

**Table:**

O1—H1	0.878 (13)	C3—C4	1.376 (3)
O1—C5	1.376 (2)	C4—H4	0.9300
N1—H1A	0.879 (12)	C4—C5	1.406 (3)
N1—C1	1.373 (3)	C5—C6	1.376 (3)
N1—C2	1.376 (3)	C6—H6	0.9300
N2—H2A	0.894 (12)	C6—C7	1.399 (3)
N2—H2B	0.914 (13)	C7—C8	1.438 (3)
N2—C10	1.473 (3)	C8—C9	1.501 (3)
C1—H1B	0.9300	C9—H9A	0.9700
C1—C8	1.358 (3)	C9—H9B	0.9700
C2—C3	1.391 (3)	C9—C10	1.518 (3)
C2—C7	1.412 (3)	C10—H10A	0.9700
C3—H3	0.9300	C10—H10B	0.9700
			
C5—O1—H1	109.1 (19)	C5—C6—H6	120.5
C1—N1—H1A	124.8 (16)	C5—C6—C7	119.01 (16)
C1—N1—C2	108.64 (16)	C7—C6—H6	120.5
C2—N1—H1A	126.4 (16)	C2—C7—C8	107.00 (17)
H2A—N2—H2B	113 (2)	C6—C7—C2	119.27 (17)
C10—N2—H2A	109.3 (16)	C6—C7—C8	133.72 (17)
C10—N2—H2B	108.5 (18)	C1—C8—C7	106.30 (17)
N1—C1—H1B	124.7	C1—C8—C9	127.65 (19)
C8—C1—N1	110.65 (19)	C7—C8—C9	126.01 (17)
C8—C1—H1B	124.7	C8—C9—H9A	109.0
N1—C2—C3	131.04 (18)	C8—C9—H9B	109.0
N1—C2—C7	107.41 (16)	C8—C9—C10	112.92 (16)
C3—C2—C7	121.54 (18)	H9A—C9—H9B	107.8
C2—C3—H3	121.0	C10—C9—H9A	109.0
C4—C3—C2	118.04 (17)	C10—C9—H9B	109.0
C4—C3—H3	121.0	N2—C10—C9	111.17 (17)
C3—C4—H4	119.4	N2—C10—H10A	109.4
C3—C4—C5	121.14 (18)	N2—C10—H10B	109.4
C5—C4—H4	119.4	C9—C10—H10A	109.4
O1—C5—C4	116.80 (17)	C9—C10—H10B	109.4
C6—C5—O1	122.29 (17)	H10A—C10—H10B	108.0
C6—C5—C4	120.90 (17)		
			
O1—C5—C6—C7	177.03 (16)	C3—C2—C7—C6	2.9 (3)
N1—C1—C8—C7	0.2 (2)	C3—C2—C7—C8	−178.28 (17)
N1—C1—C8—C9	−177.44 (19)	C3—C4—C5—O1	−176.50 (17)
N1—C2—C3—C4	178.8 (2)	C3—C4—C5—C6	2.8 (3)
N1—C2—C7—C6	−178.03 (16)	C4—C5—C6—C7	−2.3 (3)
N1—C2—C7—C8	0.8 (2)	C5—C6—C7—C2	−0.5 (2)
C1—N1—C2—C3	178.3 (2)	C5—C6—C7—C8	−178.96 (18)
C1—N1—C2—C7	−0.7 (2)	C6—C7—C8—C1	178.0 (2)
C1—C8—C9—C10	113.0 (2)	C6—C7—C8—C9	−4.4 (3)
C2—N1—C1—C8	0.3 (3)	C7—C2—C3—C4	−2.4 (3)
C2—C3—C4—C5	−0.5 (3)	C7—C8—C9—C10	−64.2 (3)
C2—C7—C8—C1	−0.6 (2)	C8—C9—C10—N2	−61.9 (2)
C2—C7—C8—C9	177.08 (18)		

### Hydrogen-bond geometry (Å, º)

**Table:**

*D*—H···*A*	*D*—H	H···*A*	*D*···*A*	*D*—H···*A*
O1—H1···N2^i^	0.88 (1)	1.77 (1)	2.636 (2)	170 (3)
N1—H1*A*···O1^ii^	0.88 (1)	2.10 (1)	2.967 (2)	169 (2)
N2—H2*B*···O1^iii^	0.91 (1)	2.19 (1)	3.092 (3)	168 (2)

Symmetry codes: (i) −*x*+3/2, −*y*+1, *z*−1/2; (ii) −*x*+3/2, −*y*, *z*+1/2; (iii) −*x*+2, *y*+1/2, −*z*+1/2.
